# Efflux Pump Mediated Antimicrobial Resistance by Staphylococci in Health-Related Environments: Challenges and the Quest for Inhibition

**DOI:** 10.3390/antibiotics10121502

**Published:** 2021-12-07

**Authors:** Abolfazl Dashtbani-Roozbehani, Melissa H. Brown

**Affiliations:** College of Science and Engineering, Flinders University, Bedford Park, SA 5042, Australia; abolfazl.dashtbaniroozbehani@flinders.edu.au

**Keywords:** antimicrobial resistance, bacterial multidrug efflux pumps, staphylococci, efflux pump inhibitor

## Abstract

The increasing emergence of antimicrobial resistance in staphylococcal bacteria is a major health threat worldwide due to significant morbidity and mortality resulting from their associated hospital- or community-acquired infections. Dramatic decrease in the discovery of new antibiotics from the pharmaceutical industry coupled with increased use of sanitisers and disinfectants due to the ongoing COVID-19 pandemic can further aggravate the problem of antimicrobial resistance. Staphylococci utilise multiple mechanisms to circumvent the effects of antimicrobials. One of these resistance mechanisms is the export of antimicrobial agents through the activity of membrane-embedded multidrug efflux pump proteins. The use of efflux pump inhibitors in combination with currently approved antimicrobials is a promising strategy to potentiate their clinical efficacy against resistant strains of staphylococci, and simultaneously reduce the selection of resistant mutants. This review presents an overview of the current knowledge of staphylococcal efflux pumps, discusses their clinical impact, and summarises compounds found in the last decade from plant and synthetic origin that have the potential to be used as adjuvants to antibiotic therapy against multidrug resistant staphylococci. Critically, future high-resolution structures of staphylococcal efflux pumps could aid in design and development of safer, more target-specific and highly potent efflux pump inhibitors to progress into clinical use.

## 1. Introduction

Antimicrobial resistance (AMR) represents a public health concern worldwide and has been described as a “slow-moving tsunami” [1]. Undoubtedly, the selective pressure exerted by the excessive and indiscriminate use of antimicrobial agents such as antibiotics and sanitisers to kill or inhibit various bacterial pathogens has accelerated the emergence and spread of AMR among bacteria [2,3]. Bacteria have developed countermeasures to protect themselves against external stresses such as antimicrobials. In the presence of lethal stress, while the majority of bacterial cells perish, a subpopulation of bacterial cells known as persister cells can survive and resume growth after relief of the imposed lethal stress [4,5]. Staphylococcal bacteria in particular can adopt alternative lifestyles including a phenotype known as “small colony variants” [6] and biofilm formation [7], which can house such persister cells. These specific lifestyles facilitate staphylococci to be more resistant to antimicrobials.

The spread of resistant bacteria from the clinic to the environment (and vice-versa) is increasing [8], causing a growing and worrying threat to public health worldwide. According to the ‘One Health’ concept, the health of humans, animals and the environment is interconnected and the spread of AMR in any of these domains poses a risk to human health. This necessitates global innovative interventions to prevent and manage AMR [9,10]. An alarming situation is when bacteria exhibit non-susceptibility to three or more agents from different antimicrobial classes, which are then defined as multidrug resistant (MDR) bacteria [11]. Infections caused by MDR bacteria are becoming significantly harder to treat causing increases in the length of stay in hospitals, in addition to economic and social costs and mortality [12]. According to the World Health Organisation (WHO) [13], we are heading for a post-antibiotic era, where common infections and small injuries may again be fatal if we fail to act urgently against the rapid spread of AMR. Antimicrobial stewardship (restricting the use) has been a challenge during the ongoing COVID-19 pandemic, with most hospitalised COVID-19 patients given antibiotics as a prevention against secondary bacterial infection [14]. This substantially increased usage, together with increased application of sanitisers and disinfectants globally, creates conditions to further accelerate the development and spread of resistant bacteria and it is therefore anticipated to worsen the AMR threat in the coming years [14,15]. It is worth noting that there is currently no stewardship for biocides [16]. Widespread use of biocides, particularly benzalkonium and chlorhexidine, can contribute to development of biocide tolerance, which has also been found to be correlated with cross-resistance to antibiotics in bacteria including *Staphylococcus aureus* [17,18].

The identification of new antimicrobial drugs remains a top medical priority given the increasing rate of infections caused by MDR bacteria. This situation has been exacerbated by the lack of interest in the discovery of new antibiotic drugs. Indeed, the new antimicrobial drug discovery pipeline has shrunk since the golden age of antibiotic discovery (1950s–1970s) [19]. Today large pharmaceutical companies seem to have little enthusiasm to develop and deliver a new class of antibiotics to clinic mainly due to the low return of investment in the last few decades. Multiple factors, including short-lived efficacy before the first resistant bacterial strains start to appear (1–4 years) and demanding regulatory requirements, are the main reasons for the lack of investment on the development of novel antimicrobial drugs [20,21]. Therefore, there is a pressing need to identify alternative ways to safeguard the effectiveness of the currently used antimicrobials for the control of bacterial infections [22]. One such orthogonal strategy complimentary to new antimicrobial discovery is the development of nonantimicrobial compounds, termed antibiotic adjuvants, that can diminish the emergence of resistance. These compounds can be used in combination with existing antibiotics to enhance the activities of already approved antibiotics to which resistance has developed [23,24].

Staphylococci are among the most important human pathogens and are well known for their ability to rapidly develop resistance to a broad range of antimicrobial compounds [25]. Additionally, staphylococci possess a remarkable diversity of resistance mechanisms which cause the increasing multidrug resistance seen among these bacteria [26]. Among the resistance mechanisms behind MDR staphylococci, active efflux pumps play a key role in the development of AMR in these bacteria by actively extruding antimicrobials from the cell [27]. The growing threat of efflux pump mediated AMR in staphylococci has driven research into identification of antimicrobial adjuvants. This is required to safeguard the continued utility of anti-staphylococcal drugs and simultaneously decrease the selection of resistant mutants via multidrug efflux. One such promising area of research is the identification and development of efflux pump inhibitors (EPIs). These antimicrobial adjuvants can potentiate a co-administered antibiotic through inhibition of staphylococcal efflux systems [28].

This review aims to present the current state of knowledge surrounding efflux-mediated antimicrobial resistance in staphylococci with their clinical implications and summarise recent updates on EPIs with a view to aiding the development of an effective adjuvant therapy to overcome efflux-related resistance in staphylococci.

## 2. Staphylococcal Infections as a Global Health Problem Requiring Urgent Attention

Staphylococci are frequent bacterial colonisers of the mucous membranes and skin of humans and other mammals [29]. Staphylococcal species contain two main groups, the coagulase-negative (CNS) and -positive staphylococci, based on their ability to produce the enzyme coagulase [30]. *Staphylococcus epidermidis*, a CNS, and *S. aureus*, a coagulase-positive species, are amongst the most frequently isolated nosocomial pathogens [30,31]. *S. epidermidis* is less virulent than its cousin *S. aureus* and only rarely causes life-threatening infections, usually in immunocompromised patients [32]. However, *S. epidermidis* represents the most common opportunistic pathogen involved in hospital-associated infections related to implantable medical devices [29,33]. Such infections have become increasingly concerning in the healthcare field as they can be extremely difficult to treat due to biofilm formation. Biofilms are more resistant than planktonic cells to antibiotics and host immune response [34]. *S. epidermidis* can also function as a reservoir for virulence and antibiotic resistance genes that can be transferred to *S. aureus*, augmenting the pathogenicity and antibiotic resistance of this more dangerous pathogen [29]. Being the most virulent staphylococcus species, the well-studied pathogen *S. aureus* is responsible for a wide spectrum of human infections ranging from minor skin and soft tissue abscesses to major fatal necrotising pneumonia, meningitis, infective endocarditis, sepsis, osteomyelitis, toxic shock syndrome as well as infections related to implanted medical devices [35,36]. Staphylococci are successful pathogens in establishing both acute and chronic infections in different human body sites due to their ability to efficiently regulate a wide arsenal of virulence factors and immune evasion mechanisms [31].

## 3. Development and Dissemination of Multidrug Resistance in Staphylococci

*S. aureus* represents the most notorious superbug among Gram-positive bacteria due to its ability to develop resistance to antibiotics [37]. Historically, penicillin was used to treat *S. aureus* infections. However, by the mid-1940s, penicillin-resistant *S. aureus* strains began to appear in hospitals only a few years after its introduction into clinical practice. Within a decade, penicillin-resistant strains spread to the community and reached pandemic levels [25,38]. Resistance to penicillin emerged through the acquisition of the *blaZ* gene. This gene encodes β-lactamase, which hydrolyses the β-lactam ring of penicillin and thus inactivates its antimicrobial activity [39]. To circumvent the problems associated with penicillin resistance, second generation semi-synthetic penicillins, which include methicillin and oxacillin, were introduced in 1959. However, resistance to methicillin was first reported in the UK in 1961, giving rise to methicillin-resistant *S. aureus* (MRSA) [40,41]. This resistance phenotype was 20 years later identified to be due to the acquisition of a *mecA* gene that encodes the low affinity penicillin binding protein (PBP) 2a, enabling the bacterium to maintain cell wall synthesis, while other PBPs are inhibited by β-lactam antibiotics [25].

*S. aureus* is highlighted as a globally pervasive pathogen associated with rising AMR [42,43,44]. Furthermore, this ‘superbug’ has garnered substantial attention from public health researchers and health policy makers due to significant morbidity and mortality caused by MDR *S. aureus* infections in the hospitals or in the community settings globally [45,46,47]. For example, MRSA strains, which are resistant to all β-lactam antibiotics, were once largely confined to hospitals but recently have been increasingly isolated from community infections [48,49,50]. These community-associated MRSA (CA-MRSA) strains have been rapidly spreading in diverse communities throughout the world since the 1990s with reports of outbreaks in several countries [51,52]. In addition to its dissemination, CA-MRSA can cause complicated infections such as infective endocarditis and sepsis, whose incidence were rare before the emergence of CA-MRSA [47,53].

In addition to β-lactam antibiotics, *S. aureus* exhibits resistance to numerous other antibiotics such as vancomycin, erythromycin, gentamicin, and oxacillin. This has resulted in increasingly limited antibiotic choices for the treatment of cognate infections [47,53,54]. A notable example of this phenomenon is the development of vancomycin resistance in *S. aureus*. Vancomycin, a glycopeptide antibiotic that inhibits cell wall biosynthesis, has been used as the drug of choice for the treatment of MRSA infections since the 1980s; however, *S. aureus* strains exhibiting decreased susceptibility to vancomycin, known as vancomycin-intermediate and vancomycin-resistant MRSA (VISA and VRSA, respectively) have emerged and are now compromising successful therapy [55,56,57,58]. The biochemistry behind the development of resistance in this pathogen is discussed in the following section.

## 4. Molecular Mechanisms of Antimicrobial Resistance in Staphylococci

Technical advances have gradually unveiled the molecular mechanisms of bacterial AMR. Bacterial resistance can emerge via two major genetic pathways: (i) spontaneous mutations/deletions in the bacterial chromosome which independently result in resistance to some antimicrobials (e.g., *S. aureus* resistance to quinolones, linezolid and daptomycin) [59,60]; and (ii) acquisition of foreign DNA, referred to as ‘mobile genetic elements’ (MGEs), through either horizontal or vertical gene transfer. MGEs include plasmids, transposons, bacteriophages, insertion sequences, integrons, pathogenicity islands, and chromosomal cassettes that can disseminate a variety of pre-existing antimicrobial resistance determinants across bacterial genomes [61,62,63,64]. While plasmids are mostly extrachromosomal, other MGEs have the ability to integrate into the host DNA [62,65]. MGEs contribute to the high-level emergence of MDR strains [61,66]. For example, *S*. *aureus* resistance to methicillin and vancomycin was developed through the acquisition of the Staphylococcal Chromosomal Cassette *mec* harbouring the mecA gene [67] and the Enterococcal *vanA* operon residing on a conjugal plasmid [68,69], respectively.

AMR can be mediated by the following biochemical mechanisms: (i) enzymatic modification of the antimicrobial binding site to decrease the affinity for the antimicrobial (e.g., resistance to methicillin by PBP 2a); (ii) enzymatic inactivation/modification of the antimicrobial (e.g., resistance to β-lactam antibiotics by production of β-lactamases); (iii) bypassing the metabolic pathway to avoid antimicrobial action (e.g., resistance to trimethoprim-sulphamethoxazole by production of an insensitive dihydrofolate reductase); (iv) sequestration of the antimicrobial to protect the target (e.g., resistance against host defence antimicrobial peptides such as α-defensins by secretion of staphylokinase); and (v) increased expression of efflux pumps to extrude antimicrobial molecules (e.g., resistance to fluoroquinolones by the NorA efflux pump) [70,71,72,73,74]. Central to the aforementioned resistance mechanisms is the expression of active efflux pumps, which act as the first line of defence against antimicrobials in staphylococci [27], and is discussed in the following sections.

## 5. Efflux-Mediated Antimicrobial Resistance: Clinical Implications

Efflux pumps are ubiquitous transport proteins which are distributed in the cytoplasmic membrane of bacteria, archaea and eukaryotes [75]. Efflux-mediated drug resistance has increasingly continued to challenge the chemotherapy of bacterial infections and human cancers [76,77]. Several studies have highlighted the contribution of multidrug efflux pumps in the formation of bacterial persisters [78]. Multidrug efflux genes were found to have significantly higher expression levels in persisters compared to efflux knockout strains which showed dramatically reduced expression [79,80]. Efflux pumps enable bacteria to survive for a period of time, increasing the probability of spontaneous mutations which cause high-level resistance to specific antimicrobials to develop. Thus, the activity of efflux pumps can contribute to the establishment of other resistance mechanisms [81,82].

Bacteria essentially utilise efflux pumps as a natural defence mechanism by virtue of their ability to expel a wide variety of toxic compounds in the environment which lowers their concentration inside the cell to sub-toxic levels [41,83]. Drug efflux pumps are considered as an effective resistance mechanism in bacterial pathogens including *S. aureus* to cope with the diverse range of antimicrobials used clinically to treat infections or as antiseptics and disinfectants to reduce bacterial load [18]. Some bacterial efflux pumps are substrate-specific since they recognise and expel a single substrate or its closely-related derivatives (e.g., tetracycline-specific pumps), whereas MDR efflux pumps recognise and export a broad spectrum of structurally-unrelated substrates [84]. In other words, substrate polyspecificity (or promiscuity toward substrates) is the characteristic feature of MDR efflux proteins [85].

In addition to antimicrobial resistance, efflux pumps in bacteria play numerous vital physiological roles including extrusion of endogenous noxious metabolites, host-derived antimicrobials and virulence factors, suggesting that the synthetic antimicrobials could be “accidental substrates” of these membrane transport proteins [86,87,88]. There is growing evidence from many studies that highlight the contribution of efflux pumps towards bacterial biofilm formation [89], particularly in several important pathogenic species of bacteria such as *S. aureus*, *Acinetobacter baumannii*, *Escherichia coli*, and *Pseudomonas aeruginosa*. These bacteria can form biofilms on medical devices and cause biofilm infections, which constitute clinical challenges [90,91].

The expression levels of efflux pump genes are generally low under ordinary environmental conditions due to strict control of multiple regulators [92,93,94]. Notably, hospital-associated bacteria, including *S. aureus*, that use efflux pumps for antimicrobial resistance often express such pumps constituently at higher levels as a result of regulatory mutations, either in the promoter region of the efflux pump or in its regulator gene [83,95,96]. This provides a piece of evidence in favour of the proposition that efflux pumps have primordial physiological functions distinct from antimicrobial resistance but have been fortuitously exploited for antimicrobial resistance roles in bacterial pathogens under intense antimicrobial selective pressures in hospital environments [83].

The use of antimicrobial susceptibility measurements (such as the minimum inhibitory concentration (MIC)) is a common method to reveal differences in antimicrobial efflux activity. Bacteria with higher efflux pump expression are less susceptible to selected antimicrobials compared to those with lower efflux pump expression. However, reflecting the limited sensitivity of this method, other methods have been developed to specifically identify resistance mediated by an efflux pump in bacterial cells [97]. Basically, these methods often utilise a fluorescent molecule known to be a substrate of the efflux pump under investigation. Some directly measure the efflux activity via assessing the amount of a fluorescent substrate that is expelled while others indirectly measure the activity by assessing accumulation of a substrate inside the cell [97,98,99].

## 6. Major Classes of Bacterial Efflux Pumps

**Figure 1:**
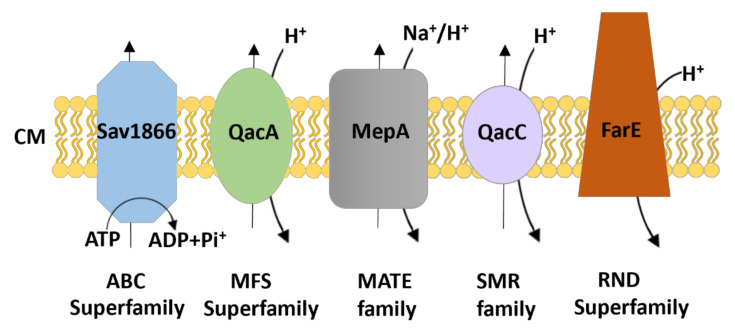
Schematic representation of the families/superfamilies of multidrug exporters in staphylococci. Each transport system is depicted as a distinct shape and colour along with the energy source for driving substrate export (i.e., ATP hydrolysis for the ABC superfamily and electrochemical energy stored in the ion gradient [H^+^/Na^+^] for the others). The transporters classified within the ATP-binding cassette (ABC), major facilitator superfamily (MFS), multidrug and toxic compound extrusion (MATE), small multidrug resistance (SMR), and resistance-nodulation division (RND) family commonly expel their substrates across the cytoplasmic membrane (CM). Examples of *S. aureus* transporters are included.

Bacterial drug efflux pumps are currently classified into seven membrane protein families based on a number of properties including protein sequence homology, structural conformation of the protein (the number of transmembrane-spanning (TMS) regions they possess), the energy source used (ATP hydrolysis or the ion electrochemical gradient), and substrate specificity. These families are: (1) the ATP-binding cassette (ABC) family; (2) the major facilitator superfamily (MFS); (3) the small multidrug resistance (SMR) family; (4) the proteobacterial antimicrobial compound efflux (PACE) family; (5) the multidrug and toxic compound extrusion (MATE) family; (6) the resistance-nodulation division (RND) family; and (7) the *p*-aminobenzoyl-glutamate transporter (AbgT) family [100]. While ABC transporters utilise the energy produced by ATP hydrolysis, other transporters depend on the electrochemical energy stored in the ion gradient, typically the proton motive force (pmf), as the driving force for the extrusion of their substrates. MATE family proteins can also use the sodium membrane gradient as the source of energy [101]. Multidrug efflux pumps identified in *S. aureus* belong to the ABC, MFS, MATE or SMR superfamilies/families of membrane proteins (Figure 1) and are discussed in the following sections. Interestingly, a new RND type efflux pump, FarE, from *S. aureus* has been identified to transport antimicrobial fatty acids found on the skin and nasal secretions [102]. Efflux systems that belong to the RND family are usually composed of three proteins. However, here FarE seems to be the only identified component of the efflux system in the staphylococcal cell envelope and may not form a tripartite assembly with partner proteins due to lack of the outer membrane. 

## 7. Prototypical Characterised Efflux Pumps in Staphylococci

Technological advances in genome analysis and bioinformatics have identified a large number of genes encoding putative efflux pumps in bacteria (http://www.membranetransport.org/) (accessed on 26 October 2021); however, the majority of these remain experimentally uncharacterised. More than 15 efflux pumps have been described in *S. aureus* thus far. These efflux pumps are encoded either on the chromosome or on plasmids [27,103]. In combination, these drug transporters potentiate resistance to a wide spectrum of unrelated antibiotics, such as tetracyclines, macrolides, and quinolones, as well as a vast array of biocides, including quaternary ammonium compounds (QACs), biguanidines and diamidines. Table 1 summarises the information of the prototypical efflux pumps within *S. aureus*.

The SMR transporters, as implied by their name, are small efflux pump proteins approximately 100–130 amino acids in length. They consist of only four TMS and are carried on plasmids. The *S. aureus* SMR transporter identified thus far is QacC (also known as Smr, QacD or Ebr) [83,136,137]. The QacC multidrug transporter (107 amino acids) is composed of four TMS [145] and predicted to form dimers in the bacterial membrane [136,146]. Similar to the prototypical SMR protein EmrE from *E. coli*, QacC has been proposed to function as a homo-oligomer [93,147,148]. Mutagenesis and functional assessments have shown that the residue E13 in TMS 1 of QacC is essential for resistance to benzalkonium. This residue, which is highly conserved in SMR members, is part of the both substrate and proton binding site of QacC [149]. In addition, highly conserved residues Y59 and W62 in TMS 3 were found to be functionally important as substitutions resulted in a significant reduction in the ability of QacC to confer resistance to benzalkonium and other compounds [145]. The *qacC* gene [130,150] can be carried on small rolling-circle replicating plasmids [151,152] and large low-copy-number multiresistance plasmids; its promoter is known to differ between these plasmids systems [130]. QacC expression is not modulated by a transcriptional regulator [141] and currently there is no high-resolution structure available for QacC protein. Similar to QacA, QacC confers resistance to some QACs (benzalkonium and cetrimide) in staphylococcal strains [145,153], but it does not provide resistance against chlorhexidine (CH) [154,155]. It has been shown that *S. aureus* isolates carrying both *qacA* and *qacC* have the potential to survive exposure to a higher dose of QACs compared to those carrying only *qacA* [156,157]. Such *S. aureus* strains with elevated resistance to QACs can lead to infections in the hospital necessitating infection control measures to prevent their transmission [157].

Proteins of the MATE family range from 400 to 550 amino acid residues. MATE transporters possess 12 TMS and are chromosomally-encoded [83,158]. The MepA efflux pump is the only MATE transporter found in the staphylococcal chromosome [113,114,159]. MepA (451 amino acids) has 12 TMS and presents 21% amino acid identity to the MATE transporter NorM from *Vibrio parahaemolyticus* [27,113,160]. MepA is associated with a MDR phenotype in clinical *S. aureus* isolates, mediating low-level resistance to several monovalent and bivalent biocides and dyes [161], as well as to tigecycline [162], a glycylcycline antibiotic. Like the MFS transporter NorA, MepA is an important fluoroquinolone efflux system in *S. aureus* that expels hydrophilic fluoroquinolone antibiotics such as norfloxacin and ciprofloxacin. Nonetheless, these antibiotic compounds are weak substrates of MepA but strong substrates for NorA [113,114,160]. In silico modelling of MepA has revealed a probable expansive central binding cavity that represents a substrate translocation pathway similar to other multidrug binding proteins, such as AcrB [160]. Site-directed mutagenesis studies have identified that residues S81, E156, A161, D183, M291, and A302 within the cavity are functionally important [160]. Expression of *mepA* is regulated by MepR, a member of the MarR family of transcriptional repressors encoded immediately upstream of *mepA*. MepR dimers bind to inverted repeats contained within operator regions upstream of *mepR* and *mepA*. The *mepR* operator contains one inverted repeat, whereas the *mepA* operator contains two such MepR binding sites. Thus, MepR reveals higher affinity binding towards the *mepA* operator site [113,159,163]. MepR is substrate responsive and represses both *mepA* and its own gene *mepR*. In the presence of MepA substrates, MepR binding to each of the *mepA* and *mepR* operator sites is essentially abrogated in a different manner as a result of a conformational change in MepR, with a markedly reduced binding affinity for the *mepA* operator site [113,159,163].

Transporters of the ABC family have a structure that consists of four domains; two transmembrane domains and two highly conserved nucleotide-binding domains [164]. The multidrug ABC exporters Sav1866 and AbcA from *S. aureus* are chromosomally-encoded and contain a transmembrane and nucleotide-binding domain fused together that dimerise to form the complete transporter [107,165]. *S. aureus* Sav1866 is a homologue of the human ABC transporter P-glycoprotein that causes MDR in cancer cells [108,166]. Functional studies have found that Sav1866 can transport diverse substrates such as ethidium, Hoechst 33,324, tetraphenylphosphonium, verapamil and vinblastine, which are also known P-glycoprotein substrates [108,167]. Sav1866 protein is one of the best-studied bacterial multidrug ABC transporters, whose three-dimensional crystal structure was determined at 3.0 Å resolution [166]. The Sav1866 structure revealed a homodimeric protein of 12 TMS where nucleotide-binding domains exhibited a ‘head-to-tail’ arrangement with a shared interface forming two ATP-binding and hydrolysis sites [107,166]. The plasmid-encoded MsrA efflux pump of *S. aureus* contains 488 residues with two putative ATP-binding domains but no hydrophobic membrane spanning domains [168]. Similar to MsrA, plasmid-mediated Vga proteins from *S. aureus* are ABC transporters that have been characterised with no transmembrane domains [112].

The MFS represents the largest and most diverse family of membrane transporters found ubiquitously in all domains of living organisms [89,169]. Proteins of the MFS family range from 350 to 600 amino acid residues and commonly possess 12 or 14 TMS [83,103]. MFS transporters that function as drug and multidrug efflux pumps can be sub-grouped into three well-characterised drug:H^+^ antiporter (DHA) families, namely DHA1, DHA2 and DHA3 [83,100,170]. DHA1 and DHA3 families have been shown experimentally to have 12 TMS [100,171], whereas DHA2 family transporters such as QacA, QacB, and TetA(K) from *S. aureus* [93,127], TetA(L) from *Bacillus subtilis* [172], EmrB from *E. coli* [173], and LfrA from *Mycobacterium smegmatis* [174], have been shown to have a 14-TMS topology. Nonetheless, evolutionary studies suggested that DHA2 transporters have evolved from a 12-TMS precursor where the extra two central TMS are localised between TMS 6 and 7 [171,175,176]. The drug resistance phenotypes due to efflux in staphylococci are predominately conferred by MFS efflux pumps [177]. The genes encoding QacA, QacB and TetA(K) are primarily carried on plasmids whereas those encoding other MFS transporters are present in the chromosome (Table 1). The following sections provide an overview of well-characterised *S. aureus* MFS efflux pumps, focusing on their structural and functional aspects as well as clinical implications.

### 7.1. NorA, NorB and NorC Efflux Proteins

NorA, together with QacA, represent the best studied MFS multidrug efflux pumps found in *S. aureus* [103]. NorA was the first endogenous (chromosomally-encoded) efflux pump to be characterised in *S. aureus* [178,179]. It possesses 12 TMS with 388 amino acids and a molecular weight of 42.2 kDa [103]. In addition to hydrophilic fluoroquinolones antibiotics (e.g., norfloxacin and ciprofloxacin; treatment options against *S. aureus* infections), NorA confers resistance to a broad range of structurally different compounds including biocides (e.g., cetrimide, benzalkonium), dyes (e.g., ethidium, rhodamines), puromycin and chloramphenicol [180,181,182]. NorA is the predominant efflux pump associated with first-line response to antimicrobials in staphylococci [122,183] and is overexpressed in 43% of *S. aureus* strains, especially MRSA [184,185] strains. Due to this clinical importance, NorA has been widely studied as a target for the development of EPIs [186,187]. However, due to lack of a high-resolution structure for NorA, chemical approaches and in silico analyses (molecular docking studies) have been implemented to identify EPIs for NorA [181,188,189,190].

NorB and NorC are chromosomally-encoded efflux pumps in *S. aureus* of 464 and 462 amino acid residues, respectively [103,125,191]. NorA confers resistance to hydrophilic quinolones such as ciprofloxacin and norfloxacin, whereas NorB and NorC also confer resistance to hydrophobic quinolones such as moxifloxacin and sparfloxacin [125,192]. These three pumps, collectively known as ‘Nor efflux pumps’, have broad substrate profiles that include not only quinolones but also other antimicrobials, disinfectants, and dyes [191,193]. The efflux pump NorB has amino acid sequence identity with *S. aureus* NorA (30%), QacA (39%) and NorC (61%) [27,125]. Expression of the Nor efflux pumps in *S. aureus* is regulated by the global regulator MgrA (multiple gene regulator). MgrA appears to act as a positive regulator (activator) of *norA* expression but as a negative regulator (repressor) of *norB* and *norC* expression [123,194,195]. Increased expression of *norB* in a mouse subcutaneous abscess model showed that NorB was important for *S. aureus* survival in abscesses, suggesting a contribution of NorB in staphylococcal pathogenesis [196,197]. Recently, the X-ray structure of NorC at a resolution of 3.6 Å in complex with a single-domain camelid antibody has been determined [198]. This study demonstrated a proof-of-principle that nanobodies have the capacity to be used as EPIs for NorC and possibly other DHA2 family transporters by locking them in an outward-open conformation.

### 7.2. TetA(K) and Tet38 Efflux Pumps

In *S. aureus*, TetA(K) is a key drug efflux pump which confers high levels of resistance to the tetracycline antibiotics [133,199]. TetA(K) has 14 TMS [200,201] and is closely related to chromosome-encoded TetA(L) protein of *B.* *subtilis* [202] and Tet38 (46% identity) of *S. aureus* [192]. The TetA(K) and TetA(L) proteins were found to be multifunctional antiporters because they transport the cations Na^+^ and K^+^ across the membrane along with tetracycline and H^+^. This indicates that these efflux pumps play physiological functions in salt and alkali tolerance in addition to antimicrobial resistance [203,204]. Tet38 has a wider substrate profile mediating resistance to tetracyclines, certain fatty acids [192] and fosfomycin [134,205], a clinically used antibiotic to target the MurA protein involved in bacterial cell wall synthesis [206]. Like NorB, the Tet38 efflux pump contributes to *S. aureus* colonisation of skin and survival in the environment of an abscess [196,207]. Moreover, it has recently been demonstrated that this protein is involved in the attachment and internalisation of staphylococci into host cells via interaction with host cell receptors CD36 and Toll-Like Receptor 2 (TLR-2) [205,208].

### 7.3. QacA Multidrug Efflux Protein

QacA comprises 514 amino acids that have been shown to be organised into 14 TMS based on hydropathy studies [209] and membrane topological analyses using alkaline phosphate and β-galactosidase fusions [127]. The QacA efflux pump is the most prevalent plasmid-encoded QAC-resistance mechanism among Gram-positive bacteria. QacA is encoded by the *qacA* gene which was the first bacterial MDR gene to be reported [93,210,211]. The *qacA* gene is carried by transmissible plasmids [212] including multiresistance plasmids such as the β-lactamase and heavy-metal resistance plasmid pSK57 [213] as well as pSK1-family plasmids [155,214,215] and pSK105 in *S. epidermidis* [216]. While *qacA* is usually found in clinical isolates of *S. aureus* [127,176,217] and CNS such as *S. epidermidis* [216], *Staphylococcus saprophyticus*, and *Staphylococcus hominis* [156,218,219,220], it has also been identified in *Enterococcus faecalis* that showed increased CH resistance [221]. This suggests that the plasmid-born *qacA* gene can spread across bacterial genera [141].

QacA is able to mediate resistance to a diverse range of cationic lipophilic antimicrobial compounds belonging to different chemical classes. The one common feature of these compounds is that they are aromatic and contain a positive charge, being monovalent or bivalent cations. Monovalent cations include QACs (e.g., benzalkonium and cetrimide) and intercalating dyes (e.g., ethidium and acriflavine). Bivalent cations include diamidines (e.g., pentamidine, 4′,6-diamidino-2-phenylindole (DAPI)) and the biguanidines (e.g., CH) [100,128]. It is worth noting that QACs are the most prominent QacA substrates, such as cetrimide, benzalkonium and dequalinium, which are commonly used as antiseptics and disinfectants [128,222].

QacA exhibits significantly higher levels of resistance for a wide range of bivalent cationic substrates such as diamidines and biguanidines as opposed to its close homologue QacB. It has been postulated that QacA evolved from QacB in response to the extensive use of bivalent cations such as chlorhexidine in hospital environments [212]. MIC analysis of diamidine compounds with structural variations indicated that QacA protein interacts with bivalent cationic substrates irrespective of the interamidine linkage, side-chain alterations, or the placement of the amidine group on the aromatic ring [128]. Transport kinetic analyses of monovalent and bivalent fluorescent substrates revealed that QacA has a high affinity binding mechanism for the recognition of bivalent cationic substrates while QacB lacks such characteristics [223]. In addition to cationic antimicrobials, QacA was shown to provide resistance to thrombin-induced platelet microbicidal protein-1 (tPMP-1), a rabbit-derived cationic antimicrobial peptide [224,225]. QacA-dependent tPMP-1 resistant *S. aureus* strains were found to survive and proliferate within rabbit model of endocarditis. Moreover, tPMP resistance was shown to be an important *S. aureus* virulence factor correlated with endocarditis in humans [73,225,226]. Of note, the presence of the QacA transporter in the cell membrane of *S. aureus* strains has been found to be linked to increased membrane fluidity [227,228,229].

The expression of *qacA* has been shown to be inducible in vivo by the addition of QacA substrates. These substrates directly bind to a regulatory protein known as QacR, a TetR family transcriptional regulator, which represses expression of *qacA* [230,231]. QacR acts as a repressor of *qacA* expression by specific binding to the 28-base pair inverted repeat 1 (IR1) operator which overlaps the *qacA* promoter site, thereby preventing *qacA* transcription [232]. Induction of *qacA* expression occurs when QacA substrates bind to QacR, inducing a conformational change in the protein such that QacR releases from the promoter region allowing transcription of *qacA* to proceed [211,231,233].

In clinical practice, disinfectants (for disinfection of inanimate surfaces) and antiseptics (for disinfection of living tissues such as hand washing and skin decolonisation prior to invasive procedures) are extensively used in hospitals to prevent spread of nosocomial pathogens [234,235]. Studies have investigated the frequency of disinfectant and antiseptic resistance genes carried by *S. aureus* isolated from clinical settings in different regions of the world. Prevalence of *qacA* among *S. aureus* clinical isolates fluctuates depending on the geographic location, varying from 10 to 80% [236]. Carriage of *qacA* has been reported to be as high as 73.9% in Australia [155], 63% in Europe [237], 8.3–26.3% in the UK [157,238], and 33–61% in Asia [234,239,240,241]. An increasing trend in *qacA* prevalence has been observed in the U.S. and Asian countries [130], establishing the relevance of this resistance determinant. A recent study on clinical staphylococci isolated from Seattle Children’s Hospital identified a novel *qacA* nucleotide sequence variant [216] associated with an increased MIC to chlorhexidine (≥4 µg/mL). Varying *qacA* prevalence across studies is due to different factors, including the number of isolates screened in each study or the type of biocides used regularly, exerting different selective pressures on *S. aureus* isolates in each region [157]. As evidenced in these studies and others, increasing use of disinfectants and antiseptics has raised concerns about the emergence of resistance to biocides in *S. aureus* at a quite alarming rate both in medical settings and in the community [219,220].

## 8. Commonly Used Methodological Approaches for Evaluating Efflux Inhibition in Staphylococci

Multiple biological assays have been applied to assess EPI candidate molecules. The process of evaluating the efficacy of candidate EPIs generally starts with susceptibility assays in bacterial strains using selected antimicrobials in the absence and presence of the EPI, also known as checkerboard assays [78,242]. For these assays in staphylococci, a set of isogenic *S. aureus* strains are typically used [189,243,244]. Most studies have been performed on NorA where they include the well-established *S. aureus* SA1199B (NorA overexpressing strain) and its parent wild-type strain SA-1199 as well as SA-K1902 (*norA*-deleted strain). Test compounds that do not show growth inhibition nor to be a substrate when used alone but interact synergistically with an anti-staphylococcal agent in the efflux pump overexpressing strain compared to the wild-type and pump deleted strains are considered a potential EPI and are taken further for subsequent evaluations.

Measurement of the fluorescence of ethidium bromide (EtBr) is the most widely used method to determine how a designated EPI inhibits the transport of antimicrobial molecules out of the cell. EtBr is often used as a universal fluorescent substrate of efflux pump since active efflux is the only known mechanism of resistance of bacteria against this compound. EtBr emits intense fluorescence only when it intercalates into DNA inside the cell. In the absence of EPI, active efflux reduces the intracellular accumulation of EtBr and thereby causes a reduction of fluorescence in a fluorometric efflux assay whereas in the presence of EPI, increased fluorescence signal intensity is observed which is a positive indicator of EPI efficiency [28,78,245]. The most frequently used antibiotic compound in EPI assessment assays in staphylococci is ciprofloxacin which is a well-known substrate for the NorA efflux pump [243,246,247,248].

The fractional inhibitory concentration index (FICi) value is often used to describe the interaction between EPIs and antimicrobial compounds [249,250] and can be defined by the following formula:

FICi = FIC_antimicrobial_ + FIC_inhibitor_

$$\mathrm{Where}\;{\mathrm{FIC}}_{\mathrm{antimicrobial}}=\;\frac{\mathrm{MIC}\;\mathrm{of}\;\mathrm{antimicrobial}\;\mathrm{in}\;\mathrm{combination}\;}{\mathrm{MIC}\;\mathrm{of}\;\mathrm{antimicrobial}\;\mathrm{alone}}$$

$$\mathrm{and}\;{\mathrm{FIC}}_{\mathrm{inhibitor}}=\;\frac{\mathrm{MIC}\;\mathrm{of}\;\mathrm{inhibitor}\;\mathrm{in}\;\mathrm{combination}\;}{\mathrm{MIC}\;\mathrm{of}\;\mathrm{inhibitor}\;\mathrm{alone}}$$

FICi results are interpreted as follows: synergy (FICi  ≤  0.5), additivity or partial synergy (0.5 < FICi ≤ 1), indifference or no interaction ( 1 < FICi  ≤ 4), or antagonism (FICi  >  4) [251]. The potentiation of antimicrobial activity is often conveniently expressed as the lowest concentration of an EPI that results in *n*-fold reduction of the MIC of an antimicrobial agent [78].

## 9. In Quest of Staphylococcal Efflux Pump Inhibitors

As mentioned before, the activity of efflux pumps in extruding various antimicrobials from the cells is recognised as an important mechanism of resistance in clinical strains of staphylococci. Moreover, efflux pumps have also been linked to biofilm formation and virulence of these bacteria. The necessity to tackle the AMR menace has encouraged research into the identification and development of potent EPIs as a promising and valid strategy to block the antimicrobial extrusion, thereby restoring antimicrobial susceptibility and extending the clinical efficacy of existing established antibiotics [252,253]. Moreover, EPIs were shown to be able to significantly decrease biofilm formation and virulence in different bacteria including *S. aureus* [248,249].

There has been great interest in the search for natural or synthetic compounds that could act as EPIs to potentiate the activity of conventional antimicrobial agents which are substrates of efflux pumps in resistant bacterial strains [254]. Studies show that the combined use of EPIs (as adjuvants) with antimicrobials is expected to: (i) increase the intracellular concentration of the antimicrobials that are extruded by efflux pumps; (ii) decrease the intrinsic resistance of bacteria to antimicrobials; (iii) reverse the acquired resistance related to efflux pump overexpression; and (iv) reduce the frequency of the emergence of resistant mutants [183,255].

The inhibition of efflux pump activity can be achieved by several approaches [28,183,256]. These include: (i) modifications of the chemical structure of existing antibiotics to decrease their affinity for binding sites of the efflux pump transporter; (ii) interference with the regulation of efflux pump gene expression to reduce the production of an active efflux pump in the bacterial cell membrane; (iii) disruption in the energy source required for efflux pump activity; (iv) use of carefully designed compounds that can be inserted inside the efflux pump cavity and act as a molecular plug hindering the transport of antimicrobials; (v) prevention of efflux pump from acquiring its active conformations; (vi) reduction in ability of efflux pump to interact with its substrates (antimicrobials) by using decoy compounds that compete with substrates for the same binding site (competitive inhibition) or cause decrease in the affinity of efflux pump for its substrates (non-competitive inhibition); and (vii) use of membrane permeabilisers that increase the influx of antimicrobial agents with subsequent increase in their intracellular concentration.

For a compound to qualify as a potent EPI it must be able to meet the following criteria [78,185,257]. It must: (i) enhance the efficacy of an antibiotic to which a strain has developed resistance as a result of the drug efflux pump activity; (ii) have no effect on sensitive strains lacking the drug efflux pump; (iii) not potentiate the activity of antibiotics which are not substrates of the efflux pump; (iv) increase the accumulation and reduce the expulsion of antimicrobial compounds which are substrates of the efflux pump; and (e) not affect the proton gradient across the cell membrane.

A diverse array of novel EPI compounds that show promising reversal of the efflux-mediated resistance in staphylococci have been reported to date. Such inhibitors for in vitro use with staphylococcal bacteria have been obtained either from screening of naturally occurring plant metabolites or small synthetic molecules. These compounds are discussed in more detail elsewhere [187,258,259]; this review focuses on summarising the potent staphylococcal EPIs reported over the past decade. Moreover, the selected EPIs were found to potentiate the activity of antimicrobial(s) by inhibiting an efflux pump when tested against an antimicrobial-resistant staphylococcal strain overexpressing the cognate efflux pump. Other EPIs tested against clinical isolates of staphylococci with MDR phenotypes, which are not specified to be due to overexpression of a specific efflux pump, were excluded from this review.

Plants are the natural source of compounds (phytochemicals) with many diverse chemical structures that demonstrate various biological activities. In recent years many studies have focused on the search for effective EPIs of plant origin [184]. This is because the advantages of using plant-derived EPIs are manifold including lowered cost in extraction/production, safe (minimal or almost no toxicity to human cells), high-level activity and environmentally friendly [260,261]. Reserpine, an alkaloid extracted from the roots of *Rauwolfia serpentina*, was the first identified plant-derived EPI that can reverse NorA-mediated resistance in *S. aureus* by potentiating the activity of fluoroquinolones [262,263]. In general, reserpine is commonly used as a control reference EPI in experiments assessing inhibitory compounds against NorA and other MDR efflux pumps [264]. However, the clinical application of reserpine to enhance the activities of clinically used fluoroquinolones could not be achieved due to its neurotoxicity at the concentrations required for inhibition of NorA [265]. Table 2 provides a list of new plant-derived EPIs found in the last decade, including their chemical class and efflux pump(s) that they inhibit as well as the antimicrobials to which resistance is reversed in a staphylococcal strain overproducing the relevant efflux pumps. These EPIs are alkaloids, flavonoids, terpenoids and polyphenols, which are the major classes of phytochemicals.

Alternate studies have focused on the search for EPIs of synthetic origin [184]. The most promising synthetic EPIs against staphylococcal efflux pumps reported in the publications over the past decade are listed in Table 3 along with their structures. Synthetic EPIs are mostly based on heterocyclic scaffolds, such as indoles, quinolines, quinolones, boronic acids and flavonoids compounds [78,266]. Especially striking is the existence of structurally complex EPIs for NorA, indicating this efflux transporter is able to bind diverse and bulky molecules [78].

Despite the effectiveness of several natural and synthetic compounds that can function as an EPI, no discrete EPI compound has yet progressed beyond clinical trials, especially due to their toxicity profiles [249,267]. Therefore, further research is still required to provide a solid basis for subsequent clinical trials by designing and developing analogues of known staphylococcal EPI scaffolds or wholly new EPI compounds with even higher potency and minimal cytotoxicity.

## 10. Conclusions

Staphylococci are among the leading pathogens of humans, causing infections in hospitals and communities worldwide [5]. Efflux-mediated MDR, particularly in staphylococci, is an urgent clinical problem, rendering many of the current antimicrobials ineffective. To counter this issue, there is an urgent need to find alternative ways to maintain the sensitivity of staphylococci against the currently available antimicrobials by decreasing their MIC. Targeting of bacterial efflux pumps via EPIs holds great promise as an approach to enhance the potency of existing antibiotic treatment regimens when co-administered. The results of the studies summarised in this review suggest that various new EPIs have been found in the last decade to have a potential to be utilised as adjuvants to antibiotic therapy against MDR staphylococci. However, to date, there are still no EPIs on the market that can be used in combination with an antibiotic for treatment of infections caused by bacteria including staphylococci, mainly because of toxicity problems and/or poor pharmacokinetic properties [78]. Recent significant advancement in protein structural determination [268] gives a new hope that in the near future, high-resolution structures of staphylococcal MDR efflux pump bound to their substrate/inhibitor will likely be resolved. This foundational knowledge will be beneficial in promoting the design and development of further EPIs exhibiting more target-specific inhibition, being effective at a low dose and safer (non-toxic), which will hopefully progress into clinical use.

## Figures and Tables

**Table 1:** Characterised drug efflux pumps in staphylococci.

Family	Transporter	TMS	Gene Location	Prominent Substrates	Reference(s)
ABC	AbcA	12	Chromosome	Hydrophobic β-lactams	[104]
	MsrA	12	Plasmid	Macrolides, type B streptogramins, erythromycin	[105,106]
	Sav1866	12	Chromosome	Vinblastine, doxorubicin, Dyes (ethidium, Hoechst 33,342)	[107,108]
	VgaA	12	Plasmid	Lincosamides, streptogramin A, pleuromutilins	[109,110]
	VgaB	12	Plasmid	Pristinamycin, streptogramin A, streptogramin B virginiamycin, mikamycin, synergistin, dalfopristin	[111,112]
MATE	MepA	12	Chromosome	Fluoroquinolones (norfloxacin, ciprofloxacin, moxifloxacin), Glycylcyclines (tigecycline), QACs (benzalkonium, cetrimide), Dyes (ethidium)	[113,114,115]
MFS	FexA	14	Transposon	All phenicols	[103,116]
	LmrS	14	Chromosome	Lincomycin, Oxazolidinone (linezolid), Phenicols (chloramphenicol), QACs (tetraphenylphosphonium), Dyes (ethidium)	[117,118]
	MdeA	14	Chromosome	Fluoroquinolones (norfloxacin, ciprofloxacin), QACs (benzalkonium, dequalinium), Dyes (ethidium)	[90,119,120]
	NorA	12	Chromosome	Fluoroquinolones (norfloxacin, ciprofloxacin), QACs (benzalkonium), Dyes (ethidium, rhodamine)	[121,122]
	NorB	14	Chromosome	Fluoroquinolones (norfloxacin, ciprofloxacin, moxifloxacin), QACs (cetrimide), Dyes (ethidium)	[123,124]
	NorC	14	Chromosome	Fluoroquinolones (ciprofloxacin, moxifloxacin), Dyes (rhodamine)	[125,126]
	QacA	14	Plasmid	QACs (benzalkonium, dequalinium), Diamidines (pentamidine), Biguanidines (chlorhexidine), Dyes (ethidum, rhodamine, acriflavine)	[127,128,129,130]
	QacB	14	Plasmid	QACs (benzalkonium), Dyes (ethidium, rhodamine 6G, acriflavine)	[127,131]
	SdrM	14	Chromosome	Fluoroquinolones (norfloxacin), Dyes (ethidium, acriflavine)	[27,103,132]
	TetA(K)	14	Plasmid	Tetracyclines	[122,133]
	Tet38	14	Chromosome	Tetracyclines, certain unsaturated fatty acids fosfomycin	[134]
RND	FarE	12	Chromosome	linoleic and arachidonic acids, rhodomyrtone	[102,135]
SMR	QacC	4	Plasmid	QACs (benzalkonium, cetrimide), Dyes (ethidium)	[136,137,138]
	QacJ	4	Plasmid	QACs (benzalkonium, cetyltrimethylammonium bromide)	[139]
	QacG	4	Plasmid	Benzalkonium, ethidium	[140,141]
	QacH	4	Plasmid	Benzalkonium, ethidium, proflavine	[141,142]
	SepA	4	Chromosome	Benzalkonium, chlorhexidine, acriflavine	[143,144]

ABC: ATP-binding cassette; MATE: multidrug and toxic compound extrusion; MFS: major facilitator superfamily; RND: resistance-nodulation division; SMR: small multidrug resistance.

**Table 2:** Potent plant-derived EPIs reported over the past decade.

EPI Compound ^1^	Chemical Class	Structure	Efflux Pump(s)	Antimicrobials ^2,3^	Reference
15-copaenol	terpene	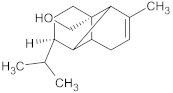	NorA	EtBr, ciprofloxacin	[184]
α-bisabolol	terpene	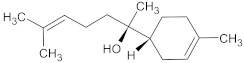	NorA, TetK	Norfloxacin, tetracycline	[269]
α-terpinene	terpene	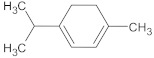	TetK	EtBr, tetracycline	[270]
benzophenanthridine	alkaloid	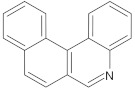	NorA	EtBr, ciprofloxacin	[271]
boeravinone B	flavonoid	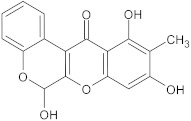	NorA	EtBr, ciprofloxacin	[190]
chalcone	flavonoid	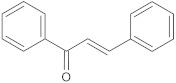	MepA, NorA	EtBr, norfloxacin	[272]
baicalein	flavonoid	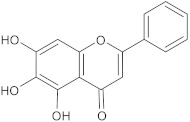	MsrA, NorA	erythromycin, ciprofloxacin	[273]
caffeic acid	polyphenol	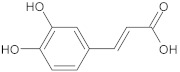	MsrA, NorA	EtBr, erythromycin, norfloxacin	[274]
caffeoylquinic acids	polyphenol	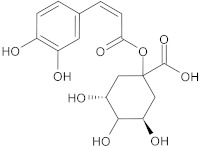	NorA	EtBr, ciprofloxacin, norfloxacin	[275]
capsaicin	alkaloid	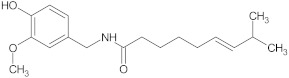	NorA	EtBr, ciprofloxacin	[276]
coumarin	polyphenol	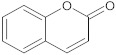	NorA	EtBr, norfloxacin	[277]
curcumin	polyphenol	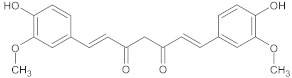	NorA	ciprofloxacin	[278]
dimethyl octanol	terpene	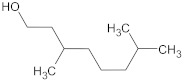	NorA	EtBr, norfloxacin	[279]
genistein	flavonoid	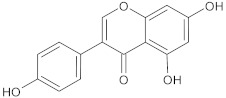	NorA	EtBr, ciprofloxacin	[280]
indirubin	alkaloid	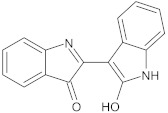	NorA	ciprofloxacin	[281]
kaempferol rhamnoside	flavonoid	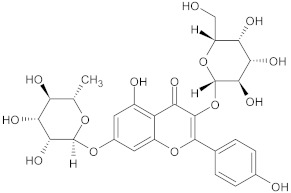	NorA	EtBr, ciprofloxacin	[282]
limonene	terpene	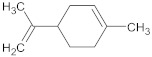	MepA	EtBr, ciprofloxacin	[283]
nerol	terpene	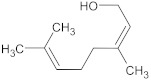	NorA	EtBr, norfloxacin	[279]
olympicin A	flavonoid	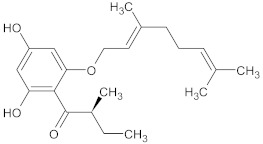	NorA	enoxacin	[284]
osthol	polyphenol	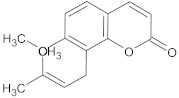	NorA	ciprofloxacin	[278]
phyllanthin	polyphenol	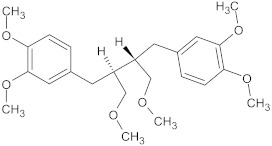	NorA	EtBr, norfloxacin	[285]
piperine	alkaloid	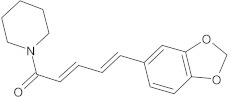	MdeA, NorA	EtBr, mupirocin, ciprofloxacin	[286,287]
tannic acid	polyphenol	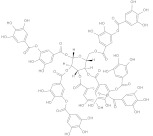	NorA	EtBr, norfloxacin	[288]

^1^ Listed EPIs potentiated the activity of antimicrobials against *S. aureus* strains overexpressing efflux pumps. ^2^ Antimicrobial compounds that are known pump substrates and were included in susceptibility and/or checkerboard assays. ^3^ EtBr: ethidium bromide.

**Table 3:** New synthetic EPI compounds reported to have synergism with various antimicrobials in staphylococci.

EPI Compound	Structure ^1^	Efflux Pump(s)	Antimicrobials ^2^	Reference
3-(1-chloro-3,4-dihydronaphthalen-2-yl) acrylic acid	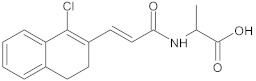	NorA	EtBr, ciprofloxacin	[289]
aglycone and 3-O-glycoside forms of flavonoids	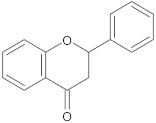	NorA	EtBr, ciprofloxacin	[290]
aminophenyl chalcone	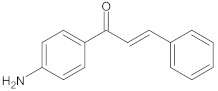	MepA	EtBr, ciprofloxacin	[291]
3-aryl-4-methyl-2-quinolones	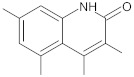	NorA	EtBr, ciprofloxacin	[292]
benzothiazine 5,5-dioxide derivatives	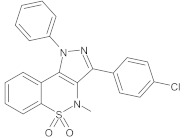	NorA	EtBr, ciprofloxacin	[293]
bis-indolic derivatives	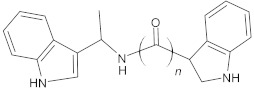	NorA	ciprofloxacin	[294]
boronic acid derivatives	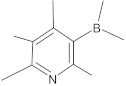	NorA	ciprofloxacin	[266]
chalcone derivatives	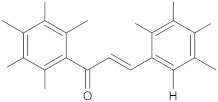	NorA	EtBr, ciprofloxacin	[295]
cinnamamide derivatives	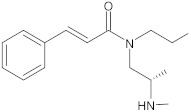	NorA	ciprofloxacin	[296]
dithiazole thione derivative	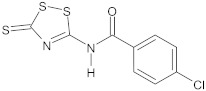	NorA	EtBr, ciprofloxacin	[297]
1-(1H-indol-3-yl)ethanamine derivatives	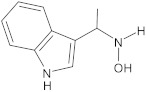	NorA	EtBr, ciprofloxacin	[298]
4-ethylpiperic acid amide derivatives	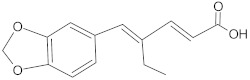	NorA	EtBr, ciprofloxacin	[299]
eugenol derivatives	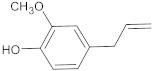	NorA	EtBr, norfloxacin	[300]
hydroxyamines derived from lapachol and norlachol	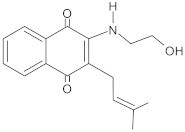	MrsA, TetK	EtBr, erythromycin, tetracycline	[301]
indole-based compounds	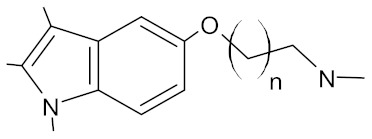	NorA	EtBr, ciprofloxacin	[181]
murucoidins	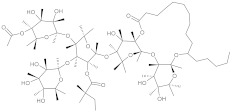	NorA	norfloxacin	[302]
1,8-naphthyridine sulfonamides	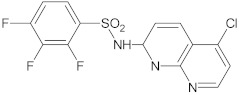	NorA	EtBr, norfloxacin	[303]
1,3,4-oxadiazole conjugates of capsaicin	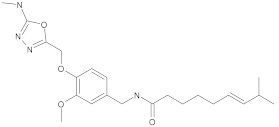	NorA	EtBr, ciprofloxacin	[304]
2-phenyl-4-hydroxyquinoline derivatives	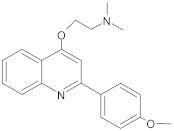	NorA	EtBr, norfloxacin	[305]
2-phenylquinoline core	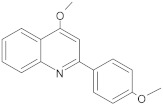	NorA	EtBr, ciprofloxacin	[243]
piperic acid amide derivatives	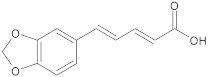	NorA	EtBr, ciprofloxacin	[299]
3-(substituted 3,4-dihydronaphthyl)-propenoic acid amides	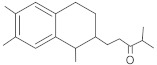	NorA	EtBr, ciprofloxacin	[306]
riparin-derived compounds	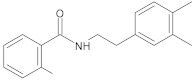	NorA	EtBr, ciprofloxacin, norfloxacin	[188]

^1^ The general structures of EPI scaffolds used for development of a series of different synthesised derivatives are presented. For details of various functional groups and linkers to a given scaffold, interested readers are referred to the original references. ^2^ EtBr: ethidium bromide.

## Data Availability

Not applicable.
